# Identifying the Resource Needs of Young People with Differences of Sex Development

**DOI:** 10.3390/jcm11154372

**Published:** 2022-07-27

**Authors:** Gina Tonkin-Hill, Chloe Hanna, Roberto Bonelli, Rowena Mortimer, Michele A. O’Connell, Sonia R. Grover

**Affiliations:** 1 Faculty of Medicine, Dentistry and Health Sciences, University of Melbourne, Melbourne, VIC 3010, Australia; chloe.hanna@rch.org.au (C.H.); rowena.mortimer@gmail.com (R.M.);; 2 Department of Gynaecology, Royal Children’s Hospital, Melbourne, VIC 3052, Australia; 3 Murdoch Children’s Research Institute, Melbourne, VIC 3052, Australia; 4 Walter and Eliza Hall Institute, Melbourne, VIC 3052, Australia; bonelli.r@wehi.edu.au

**Keywords:** differences of sex development, intersex, adolescent, young adult, disclosure, patient satisfaction, social support, psychological support systems

## Abstract

Adolescents with differences of sex development (DSD) often have complex medical, surgical, and psychological care needs and require age-appropriate resources. This cross-sectional study describes the past and current experiences of adolescents and young adults with DSD and their need for information and support. Participants aged 14–30 years with DSD diagnoses were identified, either from departmental records at the Royal Children’s Hospital (RCH), Melbourne, Australia, or from the private practice of a gynecologist linked to RCH. Anonymized data were collected from a specifically designed online survey. Of the 314 successfully traced patients, 91 (28.9%) completed the survey. Amongst respondents, older age was strongly correlated with higher levels of distress at the time of disclosure (b = 0.67, *p* < 0.001). People who reported greater understanding of their condition (b = −0.45, *p* = 0.010) and higher levels of support (b = −0.40, *p* = 0.003) identified lower levels of current distress. Respondents preferred to receive information from a specialist doctor, GP, or websites and reported information needs being highest during adolescence. Only one in four respondents recalled ever being offered psychological support. A number of perceived barriers to accessing support were identified. Our findings indicate that young people’s information and support needs may be best met by improving online resources, as well as increasing introductions to knowledgeable and appropriate primary care physicians, psychological services, and peer support groups. Further work to promote and increase engagement with psychological and peer support for those with DSD will be important.

## 1. Introduction

Differences of sex development (DSD), alternatively known as disorders of sex development or intersex variations, are a group of congenital conditions in which developmental, chromosomal, gonadal, or anatomical sex is atypical [1]. Their phenotypical overlap adds to diagnostic complexity, which can lead to a delayed diagnosis or difficulty establishing a diagnosis [2,3]. Combined with their clinical diversity, this can result in inconsistent recommendations from different specialists and fragmentation of care [4]. Historically, as diagnoses are often made in childhood, decisions regarding treatment and care, including surgery for their congenital anomalies/variations, were made before the young person could participate and give informed consent. In the past, lack of understanding and social stigma has resulted in non-disclosure of diagnosis and further negative psychosocial effects [5].

Consensus statements support multidisciplinary teams (MDTs), open communication, and full disclosure [1,6,7]. Nevertheless, adolescents and young adults with DSD face complex issues. They need relevant age-appropriate information and support to adjust and cope with their diagnosis and its potential implications [7]. Puberty, with its increased risk for mental health problems, is when adolescents with DSD may become aware of their differences [8]. Some people require pubertal induction, which, in itself, may impact on psychological well-being [7,8], while others may learn of anatomical differences that will impact fertility. This increases their information and support needs at this time.

Previous studies indicate parental care, peer relations, experiencing social acknowledgement, feelings of normality and control, and access to specialist and psychological care contribute positively to psychological well-being [4,9,10]. General practitioners (GPs) are key to care coordination, and ideally, continuous liaison should occur between GPs and the MDT. Psychological and peer support have been shown to help young people develop coping strategies, promoting positive adaptation and assisting decisions about gender, surgery, and hormone replacement [1,10]. Previous research suggests that psychological support needs to be offered repeatedly, over time [11].

At the Royal Children’s Hospital, Melbourne, Australia (RCH), children and adolescents with DSD are managed by a MDT comprising pediatric endocrinologists and surgeons (gynecology and urology), clinical geneticists, clinical ethicists, and a DSD clinical coordinator. The DSD coordinator provides primary liaison between the family and the MDT [12] and connects to online information, support groups, and psychologists in the community.

Self-reports from children and young adults about their information and support needs are required to guide optimal care planning. It is known that parents overestimate physical health while underestimating emotional health [13]. Due to the relative rarity of DSD conditions, studies to date have been limited by small sample sizes, and comparisons between different diagnoses are limited [10,14]. This study offers an insight into young people’s resource needs with a moderate sample size including three sufficiently large diagnostic groups to be compared. We aimed to examine (i) the lived experience of participants relating to disclosure of their DSD, their levels of distress, and their understanding of their variation; (ii) participants’ experience within the medical system; and (iii) participants’ age-specific preferences for information and support, including primary physician care, psychological, and peer support.

## 2. Materials and Methods

The past and current experiences of adolescents and young adults with DSD were studied between February and May 2017. The cross-sectional study included young people aged 14–30 years with DSD diagnoses, as defined in the 2006 consensus statement [1], but it excluded those with significant intellectual disability or need for an interpreter. Potential participants were identified from the electronic medical record and departmental clinical lists and recruited by a researcher with no prior contact. The age and gender/sex of non-participants were collected from their file. Giving an email address and clicking on an emailed survey link implied consent [15]. Participants under 18 years required additional parent/guardian consent. RCH Human Research Ethics Committee (HREC37003) approved the research.

Patients were surveyed by using a specifically designed questionnaire delivered online [15]. Anonymized data were analyzed by using Excel 14.0.0 and R statistical software version 3.4.0. Inference on Likert scales was performed by using linear regression models employing a Wald test. The three largest diagnostic groups were used for comparisons: MRKH (Mayer–Rokitansky–Küster–Hauser), CAH (congenital adrenal hyperplasia), and TS (Turner syndrome). Differences in rate of participation were examined by using a binomial test for diagnoses and a chi-squared test for gender/sex and age. Distress was assessed by responding “strongly agree” or “agree” to feeling worried/troubled/distressed on a 5-point Likert scale. All linear regression models were corrected for potential confounding effects, and their results are presented in terms of regression coefficient (b) and *p*-value (*p*). The *p*-values < 0.05 are considered significant.

## 3. Results

### 3.1. Demographics

Of 438 potential participants, 314 were successfully contacted. Survey links were emailed to 116 people; 91 participants completed the survey.

Table 1 displays the diagnoses, age brackets, and gender for traced participants. The participation rate for people with MRKH (47.9%) was significantly higher than the general response rate (28.9%, *p* = 0.006). Males participated at a significantly lower rate (12.6%) than females (33.3%, *p* < 0.001). The numbers of people identifying with non-binary identities were too low to compare in this group. People <18 years participated at a significantly higher rate (47.4%) than people >18 years (10.2%, *p* < 0.001).

The mean (SD) age of participants at completion of survey was 22.5(4.8) years. For the majority of their care, 58 participants reported care provided at our tertiary pediatric center, 21 in private consultations, 11 in another tertiary Australian hospital, and 1 overseas. All participants >18 years had completed high school. Two participants identified as Aboriginal. Currently, 78 respondents identify as female, 11 as male, and 2 as intersex. No participant reported previous gender incongruity; however, one “did not know” and nine did not respond. Sex was recalled as uncertain at birth in seven (8%) people, while six (7%) did not know. Of respondents, 80 were assigned female at birth, and 11 were male. Reassignment of sex occurred in two children (one male and one female), both aged <4 years.

### 3.2. Disclosure, Distress, and Understanding

Of participants, 41% were told about their DSD aged 0–4 years, 9% between 5 and 9 years, 18% between 10 and 14 years, and 31% between 15 and 19 years, while 2% did not remember. Of the 24 who were told aged 15–19 years, 6 had primary ovarian insufficiency (POI) and 16 had MRKH. The number of years (presented here as mean (SD)) since learning of their diagnosis for the largest diagnostic groups was 18.1 (5.7), 16.2 (7.3), and 9.0 (6.1) years for CAH, TS, and MRKH, respectively. Participants were first told about their DSD by their doctor (51%), their parent/guardian (34%), or other (15%).

The percentage and number of participants answering “strongly agree” or “agree” on a 5-point Likert scale to questions about distress, understanding and support is shown in Table 2. For the purposes of this study, “disclosure” refers to the time the participant learned of their DSD. Older age at the time of diagnosis correlated with greater initial distress (b = 0.67, *p* < 0.001). Adjusting for age, individuals with MRKH had significantly higher distress at disclosure compared to those with CAH (b = −2.0, *p* < 0.001) and TS (b = −2.1, *p* < 0.001). At disclosure, distress was not correlated with self-reported understanding of diagnosis (b = 0.10, *p* = 0.43).

After adjusting for age, the group of participants with Turner syndrome had significantly higher current self-reported understanding of their diagnosis than those with CAH (b = −0.56, *p* = 0.03) and MRKH (−0.56, *p* = 0.02). Greater understanding of DSD correlated positively with feeling comfortable discussing their DSD with others (b = 0.46, *p* = 0.02).

### 3.3. Information and Support Needs

Of 86 respondents, the majority preferred to access information from their specialist doctor (81%), websites (51%), and general practitioner (48%). Respondents (*n* = 64) would have liked more information on a database of knowledgeable doctors (48%), tips on explaining to a new health professional (45%) or to others (39%), links to psychological support (45%) or peer support (34%), variety of treatment options (39%), fertility (36%), body diversity (30%), sexuality/intimate relationships (27%), sex education (22%), gender identity (20%), menstruation/periods (17%), and bladder and bowel function (13%). Desire for additional information correlated with current distress (b = 0.13, *p* = 0.05). 

**Figure 1:**
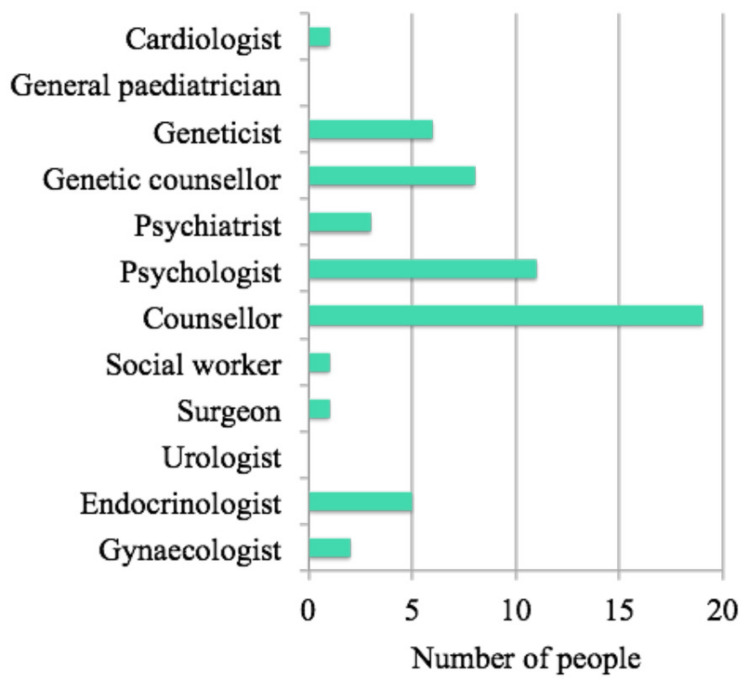
Health professionals that people would “at any stage have liked to have seen” but have not seen in the past or currently (X^2^ = 61.6, *p* < 0.0001).

Health professionals that the respondents would have liked to have seen but have not seen in the past or currently are shown in Figure 1. The majority nominated counsellor and psychologist.

**Figure 2:**
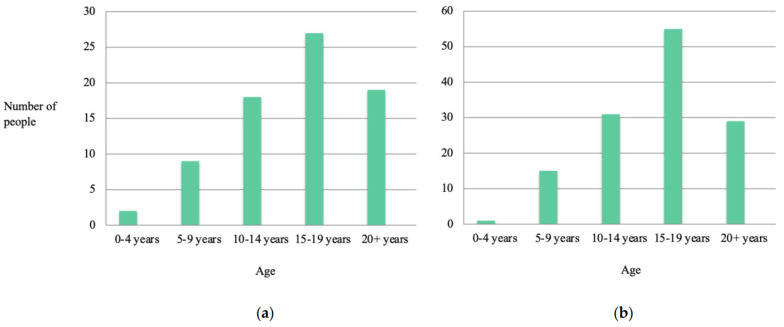
(**a**) Age at which participants would have liked to see any additional health professionals (X^2^ = 26.1, *p* < 0.0001). (**b**) Age at which participants would have liked to access additional information (X^2^ = 59.4, *p* < 0.0001).

The age at which respondents would have preferred more information and access to health professionals is shown in Figure 2. For both, the peak request occurs between 15 and 19 years of age.

Of 90 respondents, 63% discuss their DSD with a regular GP. Of those who do not (*n* = 33), 24% indicated they would like to. Related to their DSD, 49% have seen more than one GP, while 14% have never spoken to a GP. Of the 192 GPs seen by survey participants, 42% were considered helpful, 28% were considered unhelpful or lacking DSD information, and 31% were neutral or did not know. There was no correlation between distress and regular GP care (b = −0.26, *p* = 0.36). However, current distress significantly correlated with lower reported GP understanding (b = −0.37, *p* = 0.02).

Respondents (*n* = 84) felt supported by family (80%), specialist doctor (60%), friends (39%), partner (39%), GP (36%), psychologist (9%), or peer support group (7%). A minority of respondents (<5%) reported being supported by a psychiatrist, genetic counsellor, social worker, or social media. Feeling supported strongly correlated with participants feeling comfortable discussing their DSD with others (b = 0.61, *p* < 0.001).

Twenty-three (26%) participants recall being offered counselling or psychological support regarding their DSD. Participants who were offered psychological support, or who accessed it themselves, had significantly higher levels of distress at diagnosis (b = −2.64, *p* = 0.01). Of those who recall being offered psychological support, 83% did not use the service. The reasons included the following: they thought it would not help (68%), feeling they did not need it (11%), already accessing psychological care elsewhere (11%), embarrassment (5%), expense (5%), and long waiting times (5%). Of 83 respondents, 18% reported that their DSD affects their mental health, which correlated with their current DSD-related distress (b = 1.27, *p* < 0.001).

Of 18 (20%) respondents who had tried accessing peer support, 61% felt they were provided relevant information. Of these, 91% “strongly agree” or “agree” to peer support groups being helpful. Difficulty accessing peer support was reported by 50% (*n* = 9) of respondents. Respondents would prefer someone from the hospital introduced them (72%), to make contact themselves (11%), and did not know (17%). Respondents accessing peer support were significantly more distressed at diagnosis (b = 0.88, *p* = 0.02); however, this correlation disappeared when compared to current distress. Of 72 respondents (80%) who have not accessed peer support, 40% state that they did not need extra support, 38% preferred to deal with things on their own, 26% had never heard of peer support, 17% did not think it would help, 13% felt embarrassed, 1.4% were worried it was not confidential, and 10% did not know.

## 4. Discussion

This study affords important insights into the self-reported experiences and resource needs of a cohort of young people with DSD. While the majority reported high levels of support, significant opportunities for improvement are identified. Features associated with current distress included distress at disclosure, low understanding of their DSD, poor support, and lower GP understanding. Importantly for service provision, late adolescence was reported as the peak time when additional information, resources, and access to a broader range of clinicians is needed. With increasing independence, finishing school, and transitioning to adult services, this is known to be a time of change for young people [5].

This study supports the widespread recommendation for open communication and early disclosure of DSD diagnosis to enhance adaptive coping skills [1,7,16,17]. Older age at the time of disclosure correlated strongly with higher distress at disclosure. Distress may relate to the nature and implications of the diagnosis, as well as greater age-appropriate level of understanding. Higher distress at disclosure also correlates strongly with higher current distress, suggesting that earlier, more positive disclosure may prime for less distress in the future. However, for those with MRKH or POI, diagnosis is typically not apparent until adolescence, and, thus, the older age of disclosure is unavoidable.

The study found that, regardless of age at disclosure, those with a diagnosis of MRKH were more distressed than those with CAH or TS. Contributing factors may be that participants with CAH and TS learned of their diagnosis at a younger age and had longer to understand its impacts on their bodies and well-being. For people with MRKH, there is evidence that distress reduces with time from diagnosis [18], which may mirror the trajectory for those with CAH and TS reported here.

Compared to when they were diagnosed, participants’ level of distress was improved, as was their understanding of their diagnosis. Current distress did not correlate with specific DSD or age. Interestingly, while distress did not correlate with level of understanding at disclosure, current distress correlated with current DSD understanding and level of support. This highlights that, while initial distress is influenced by the type of diagnosis and age, information and support needs gain importance over time.

Some participants, across a range of conditions, did not know whether their sex at birth was uncertain. The reported rate of uncertain sex at birth (8%) was also quite low, considering the high proportion of participants with CAH. We could infer that, although participants were likely given clear information about their health history, the uncertainty of sex at birth was not emphasized. These participants did not express increased distress regarding non-disclosure.

Significant developmental changes occur during adolescence [19]. People with DSD may experience extra social and emotional challenges associated with atypical anatomy, infertility, hormonal function, and genes [20]. In our cohort, participants preferred to access DSD information from their specialist doctor, GP, and websites. The desire for more information correlates with feeling distressed. This is a cross-sectional study, so it limits our ability to imply causality. Nonetheless, our results suggest that, with improved understanding, participants may feel less distressed, more comfortable speaking with others about their DSD, and also more supported.

The importance of support from a well-informed and regular GP is identified, although participants report difficulty accessing knowledgeable GPs. Similarly, the American Academy of Pediatrics [21] states that the absence of primary care involvement leads to expensive, episodic, and fragmented care. Young people can find explaining their condition to health professionals uncomfortable or burdensome, while GPs may have infrequent experience with DSDs and have difficulty finding sufficient information. Given the associations of lower distress with feeling well informed and supported, our data support efforts to improve information and resources for GPs. As young people also endorsed the use of trusted websites for information, online resources that they can share with their GP could also be helpful. 

Stigmatization predisposes individuals to poor mental health [22]. Less than half of study participants felt comfortable discussing their DSD with others, and one-third felt unsupported. While participants reported that their main sources of support were family, specialist doctors, and GPs, they reported lower rates of support from friends and partners. It is not known if participants shared their diagnosis with friends and partners or whether they do not require their support. While current care models and advocacy groups seek to portray DSDs as part of the natural spectrum of development [23,24], there remains a general lack of awareness of DSD in society [25]. Almost half of participants would like to be linked to psychological support, and one-third would like information on peer support; these findings are similar to those of other studies [4,9,10].

While other studies have reported high rates of psychological distress [10,20], less than 20% of participants in this study reported their DSD affecting their mental health. This rate remains slightly higher than the global adolescent population [26]; however, this result concurs with previous research at RCH which found the physical and mental health of the DSD group to be similar to that of Hirschsprung and diabetes mellitus comparison groups [27]. Due to the self-selecting participation in these studies, those who were struggling may have been less likely to participate. Our study still indicates an unmet need for psychological support and perceived barriers to access. While a distressed minority recalled being offered psychological support, the majority did not. As our MDT does not include a psychologist (due to resource constraints); this may reflect genuinely low rates of referral and/or recall bias. Where offered, low rates of uptake of psychological support could also reflect participants’ fear of non-DSD-specialized care and the effort involved in explaining their condition, as community psychologists’ expertise in DSD management is often limited [20]. At our center, a DSD care coordinator, who was introduced a few years ago, provides support and resources to all children and young people and their families. The effect of this may not yet be present in this study. Our findings also support previous research recommending repetitive offers of psychological support [11]. Lastly, referrals to professionals with specific expertise are necessary where a dedicated psychologist is not part of the MDT, and, moving forward, a database of DSD-knowledgeable psychological professionals would be beneficial.

While only 20% of respondents reported seeking peer support, it was reported as helpful. Many found it difficult to make contact with peer support and stated that they would like to be introduced by the hospital. The correlation between distress and accessing peer support disappeared over time, indicating that peer support may partly decrease distress associated with diagnosis. While over a third of participants who had not accessed peer support stated that they did not need extra support, more than a quarter had never heard of a peer support group. This suggests the availability and potential benefits of peer support groups could be better promoted by the treating team and online.

Our study has a number of strengths and limitations. Adolescents and young adults were specifically targeted to explore ways to improve care pathways. The sample size was modest but relatively large compared to other DSD studies [10,20,28]. MRKH was the only group with significantly higher participation, and they reported the most distress at diagnosis. While MRKH participants were older at the time of diagnosis and may have had better recall, selection bias may have impacted this finding. Similar to other studies [10,28], the participation rates of males and of older patients were significantly lower. The number of female participants may be explained by the gynecology department’s DSD database, which includes private gynecology patients. Older patients may have been less inclined to participate due to a lower sense of connection to their affiliated treating physician or hospital [28]. No recruitment from organizations or support groups occurred.

## 5. Conclusions

Adolescents with differences of sex development often have complex medical and psychological care needs and require age-appropriate resources. This study informs future care planning and areas for improvement related to the specific information and psychosocial support needs of adolescents with DSD and their preferences for connection with support services. Low access to and uptake of psychological support and an unmet desire for information in the teenage years was identified. Importantly, people who reported greater understanding of their condition and higher levels of support had lower levels of current distress. This key finding underscores the importance of ensuring young people are well informed in relation to their bodies and the potential implications of their DSD. Preferred methods of accessing information were from a specialist doctor, GP, or websites. Barriers still exist for people wanting to connect with psychosocial supports, even when this support is offered. Our findings outline the importance of providing regular opportunities for peer and professional psychosocial supports, as well as improving individual, GP, and other healthcare professional knowledge of DSD to optimally support this potentially vulnerable cohort.

## Figures and Tables

**Table 1:** Diagnosis, age (current and at diagnosis), and gender of survey participants; 0–4 years includes “as long as I can remember”. DR = “I don’t remember”.

*n* = 91	Total	Current Age	Age at Learning of Diagnosis
		Years (Mean (SD))	Years (Mean (SD))
***Age***			
<18	16		
≥18	75		
***Gender***			
Female	78		
Male	11		
Intersex	2		
***Diagnosis***			
Congenital adrenal hyperplasia	17	21.4 (4.5)	3.5 (3.3)
Turner syndrome	18	21.7 (3.9)	6.7 (4.7)
MRKH	23	23.7 (3.2)	15.5 (2.3)
Androgen insensitivity	4	22.0 (6.1)	10.3 (6.2)
Bladder exstrophy	6	22.0 (5.8)	4.5 (3.8)
Cloacal anomalies	2	19.5 (2.5)	2 (0)
Gonadal dysgenesis	7	23.4 (4.4)	7.7 (6.8)
Primary ovarian insufficiency	7	25.6 (2.3)	16.3 (1.7)
Klinefelter syndrome	2	22.0 (0)	4.5 (2.5)
5-alpha reductase deficiency	1	17	17
Hypogonadotropic hypogonadism	1	17	12
Anorchia	2	17.0 (0)	7 (5)
VACTERL without uterus	1	27	2

**Table 2:** Percentage and number of participants answering “strongly agree” or “agree” on a 5-point Likert scale to questions on distress, understanding and support.

	%	N
*At disclosure…*		
I was given enough information	62%	44
I understood my DSD	49%	40
I felt worried/troubled/distressed	48%	38
*Currently…*		
I understand my DSD	82%	75
I feel worried/troubled/distressed	24%	22
I feel comfortable discussing my DSD with others	49%	43
I feel well supported	66%	58

Current distress correlated with higher distress at disclosure (b = 0.47, *p* < 0.001), lower self-reported understanding of their DSD (b = −0.45, *p* = 0.010), and not feeling supported (b = −0.40, *p* = 0.003). Current distress did not correlate with diagnosis or current age (b = 0.02, *p* = 0.53).

## Data Availability

The data are not publicly available to maintain participant confidentiality.
